# Protein phosphatase 4 is an essential positive regulator for Treg development, function, and protective gut immunity

**DOI:** 10.1186/2045-3701-4-25

**Published:** 2014-05-07

**Authors:** Fang-Hsuean Liao, Jr-Wen Shui, En-Wei Hsing, Wan-Yi Hsiao, Yu-Chun Lin, Yi-Chiao Chan, Tse-Hua Tan, Ching-Yu Huang

**Affiliations:** 1 Immunology Research Center, National Health Research Institutes, Zhunan, Miaoli County 35053, Taiwan; 2 Department of Pathology and Immunology, Baylor College of Medicine, Houston, Texas 77030, USA

## Abstract

**Background:**

Protein phosphates 4 (PP4), encoded by the *ppp4c* gene, is a ubiquitously expressed phosphatase that has been implicated in the regulation of cytokine signaling and lymphocyte survival; recent reports suggest that PP4 may be involved in pre-TCR signaling and B cell development. However, whether PP4 also modulates the functions of peripheral T cells has not been investigated due to the lack of a suitable *in vivo* model. Treg cells are a specialized subset of CD4 helper T cells that can suppress the proliferation of activated effector T cells. In the absence of this negative regulation, autoimmune syndromes and inflammatory diseases, such as human Crohn’s disease, will arise.

**Results:**

In this report, we generated mice with T cell-specific ablation of the *ppp4c* gene (CD4cre:PP4^f/f^) and a Foxp3-GFP reporter gene to examine the roles of PP4 in Treg development and function. Characterizations of the CD4cre:PP4^f/f^ mice showed that PP4 deficiency induced partial αβ T lymphopenia and T cell hypo-proliferation. Further analyses revealed significant reductions in the numbers of thymic and peripheral Treg cells, as well as in the efficiency of *in vitro* Treg polarization. In addition, PP4-deficient Treg cells exhibited reduced suppressor functions that were associated with decreased IL-10, CTLA4, GITR and CD103 expression. More interestingly, the CD4cre:PP4^f/f^ mice developed spontaneous rectal prolapse and colitis with symptoms similar to human Crohn’s disease. The pathogenesis of colitis required the presence of commensal bacteria, and was correlated with reduced Treg cells in the gut. Nevertheless, PP4-deficient Treg cells were still capable of suppressing experimental colitis, suggesting that multiple factors contributed to the onset of the spontaneous colitis.

**Conclusions:**

While the molecular mechanisms remain to be investigated, our results clearly show that PP4 plays a non-redundant role for the differentiation, suppressor activity and gut homeostasis of Treg cells. The onset of spontaneous colitis in the CD4cre:PP4^f/f^ mice further suggests that PP4 is essential for the maintenance of protective gut immunity. The CD4cre:PP4^f/f^ mice thus may serve as a good model for studying the interactions between Treg cells and gut commensal bacteria for the regulation of mucosal immunity.

## Background

Protein phosphatase 4 (PP4/PPX) is a ubiquitously expressed serine/threonine phosphatase that belongs to the PP2A/PP4/PP6 family [1]. Human and mouse PP4 nucleotide sequences, encoded by the *ppp4c* genes, are well-conserved with identical translated amino acid sequences, hinting an evolutionary pressure to preserve the function of PP4. Indeed, the embryonic lethality of *ppp4c*-knockout mice suggests that PP4 is essential for fetal development [2]. Initially identified as a mediator of TNFα signalings through the activation of JNK [3], PP4 is now implicated in many biological processes such as apoptosis [4], microtubule organization [5] and DNA double strand break repair [6,7]. Nevertheless, while these reports convincingly identify possible functions of PP4, their conclusions are often shadowed by the use of siRNA and chemical inhibitors that may carry off-target effects, particularly on PP2A and PP6.

To more definitively interrogate the functions of PP4 *in vivo*, we generated mice carrying a floxed *ppp4c* allele (PP4^f^) by embryonic stem cell targeting, and introduced proximal Lck promoter-driven Cre recombinase transgene (Lckcre) to mediate T cell-specific deletion of *ppp4c* (Lckcre:PP4^f/f^). Analyses of the Lckcre:PP4^f/f^ mice reveal that PP4 deficiency blocks pre-TCR signaling and induces apoptosis of immature thymocytes [2]. Recent data also show that PP4 can regulate apoptosis in primary human T cells [4]. These results thus suggest that PP4 may be an important mediator of T cell expansion and survival. Further analysis of the functions of PP4 in peripheral T cells, however, is prohibited by the absence of mature T cells in the Lckcre:PP4^f/f^ mice [2].

A specialized subset of CD4 helper cells constitutively expresses CD25 on their surface, and is termed regulatory T (Treg) cells for their ability to suppress the proliferation of neighboring T cells [8]. Treg cells develop in the thymus (known as nTreg), but can also be induced from naïve T cells *in vitro* under proper polarizing conditions (known as iTreg). The differentiation and function of Treg cells are critically enforced by the master transcription factor Foxp3 and its downstream genetic programs [9]. Recent reports, however, suggest that the lineage stability and function of Treg cells are also critically controlled by epigenetic regulations on Foxp3 and other Treg-related genes [10,11]. Regardless of how the Treg lineage is maintained, proper Treg function is pivotal for the establishment of a protective immune system, as the deficiency of *foxp3* gene ablates Treg cells and causes multiple autoimmune syndromes [12]; the deletion of *foxp3* in adult Treg cells also induces catastrophic autoimmunity [13].

Inflammatory bowel disease (IBD) is one of the human disorders that are considered to have immunopathogenesis origin [14]. IBD can be further categorized into Crohn’s disease and ulcerative colitis, in which Crohn’s disease is thought to be caused by deregulated Th1/Th17 inflammatory response, while imbalanced antibody reaction is considered to be upstream of the exacerbation of ulcerative colitis [14]. Still, non-immune components, including alterations in commensal microbiota, epithelial barrier integrity, and gut exocrine function all contribute to the onset of IBD [14,15]. With this complex nature of IBD in mind, it is not surprising that multi-pronged approaches are required to study the many aspects of IBD pathogenesis. In this regard, many spontaneous and inducible IBD mouse models have been developed to investigate the etiology of IBD *in vivo*[15], of which several reports have indicated Treg cells to be an important regulator of IBD [16-18].

To study the functions of PP4 in Treg cells, we crossed the PP4^f^ allele with CD4cre transgene (CD4cre) [19] and the Foxp3-GFP reporter knock-in gene [20] to generate the CD4cre:PP4^f/f^:Foxp3-GFP^+^ mice. Characterizations of these mice revealed important modulatory roles of PP4 on Treg differentiation, homeostasis and function. Furthermore, the spontaneous rectal prolapse and colitis that developed in the CD4cre:PP4^f/f^:Foxp3-GFP^+^ mice further indicated an immune regulatory function of PP4 for maintaining the immunological balance in the gut.

## Results

### PP4 deficiency induces partial αβ T cell lymphopenia

**Figure 1:**
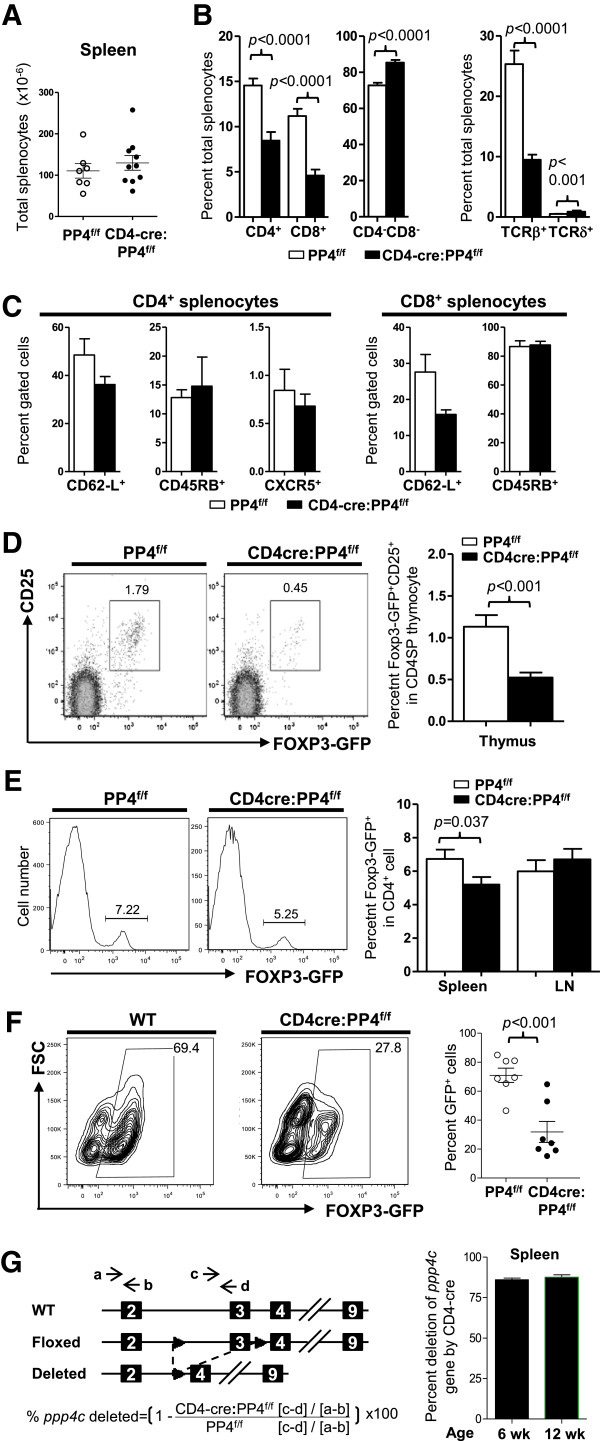
**The ablation of PP4 induces partial αβ T lymphopenia and hampers Treg differentiation *in vivo *and *in vitro*. A**, Splenocytes from 4-6 wk old mice were enumerated (*n* = 6-10). **B-C**, splenocytes were stained for various T cell markers, followed by flow cytometry analyses for the composition of CD4^+^, CD8^+^, αβ T, γδ T or CD4^-^CD8^-^ cells in total splenocytes (*n* = 7-12) **(B)**; alternatively, the expression of various lineage or activation markers on gated CD4^+^ or CD8^+^ cells was analyzed (*n* = 3-7) **(C)**. **D**, Thymocytes were analyzed for the percentages of Foxp3-GFP^+^ cells in gated CD4SP population. Representative flow cytometry results are shown (left panels). Statistical analyses results are shown (right panel; *n* = 8 ~ 12). **E**, CD4-gated splenocytes were analyzed as in A. Representative flow cytometry results are shown (left panels). Statistical analyses results are shown (Right panel; *n* = 14 ~ 19). **F**, Naïve CD4^+^CD62-L^+^ cells were MACS-purified and activated under Treg polarization condition. Cells were harvested on d 3 to analyze Foxp3-GFP expression in CD4-gated population. Representative flow cytometry results and statistical analyses are shown (*E* = 4, *n* = 7). **G**, Splenic CD4^+^Foxp3-GFP^+^ Treg cells were sorted from 6 or 12 wk old mice. DNA from these cells was analyzed by qPCR to measure the extents of *ppp4c* gene deletion (see schematics for primer design and deletion efficiency calculation; left panel). Statistical analyses results are shown (right panel; *n* = 2). See Additional file 1: Figure S1 for flow cytometry gating strategies.

Our previous analyses of the Lckcre:PP4^f/f^ mice showed that the ablation of PP4 resulted in near-complete lymphopenia in the periphery [2]. To examine whether peripheral T cells could develop normally in the absence of PP4, we analyzed 4-6 wk old CD4cre:PP4^f/f^ mice, and found that they had normal numbers of total splenocytes (Figure 1A). Closer examinations, however, revealed significant reductions in the percentages of CD4^+^ (~50% of PP4^f/f^ control) and CD8^+^ (~30% of PP4^f/f^ control) T cells (Figure 1B, left panel). These reductions were mostly attributed to the decrease in αβ T cells (Figure 1B, middle panel), and were accompanied by compensatory increases in the percentages of γδ T cells and CD4^-^CD8^-^ T cells in the CD4cre:PP4^f/f^ mice (Figure 1B, middle and right panels). To see if these reductions were limited to specific αβ T cell lineages or activation status, we further subsetted CD4cre:PP4^f/f^ αβ T cells by markers such as CD62-L, CD45RB (naïve T cells) and CXCR5 (follicular helper cells), and found that the expression of these markers in both CD4^+^ and CD8^+^ cells was similar to those from WT littermates (*p* > 0.05, Figure 1C).

### PP4 is essential for the differentiation, function and gut homeostasis of Treg cells

Treg cells are essential for maintaining a balanced immune system [21]; in addition, their importance in gut immunity has been demonstrated in adoptive transfer models [16] and in gene-deficient mice [22]. To assess the roles of PP4 in Treg development and function, we crossed the CD4cre:PP4^f/f^ mice with mice carrying the Foxp3-GFP reporter gene [20] (hereon referred as CD4cre:PP4^f/f^:Foxp3-GFP^+^ mice). Flow cytometry analyses showed that the percentage of CD25^+^Foxp3-GFP^+^ cells in CD4 single-positive (CD4SP) thymocytes was reduced by 2-fold in the CD4cre:PP4^f/f^:Foxp3-GFP^+^ mice (Figure 1D; *p* < 0.001); similar reductions were also found in splenic CD4 T cells (Figure 1E; *p* = 0.037), but not in the lymph nodes (LNs) (Figure 1E). To test whether the deletion of *ppp4c* gene impacted Treg polarization *in vitro*, WT or CD4cre:PP4^f/f^ CD4^+^CD62-L^+^ cells were activated under Treg-polarizing condition; the results showed that the induction of Foxp3-GFP^+^ cells was significantly reduced from ~70% in WT cells to ~30% in CD4cre:PP4^f/f^ cells (Figure 1F; *p* < 0.001). Lastly, qPCR was performed using genomic DNA from sorted splenic CD4^+^Foxp3-GFP^+^ cells to assess the efficiency of *ppp4c* deletion in the CD4cre:PP4^f/f^:Foxp3-GFP^+^ mice; the results showed that the *ppp4c* gene was indeed deleted in >85% of the Treg cells (Figure 1G). Western analyses of purified T cells also confirmed the deficiency of PP4 in the CD4cre:PP4^f/f^ mice (Additional file 1: Figure S2A). These findings thus suggest that PP4 is essential for the differentiation and homeostasis of Treg cells *in vivo*.

**Figure 2:**
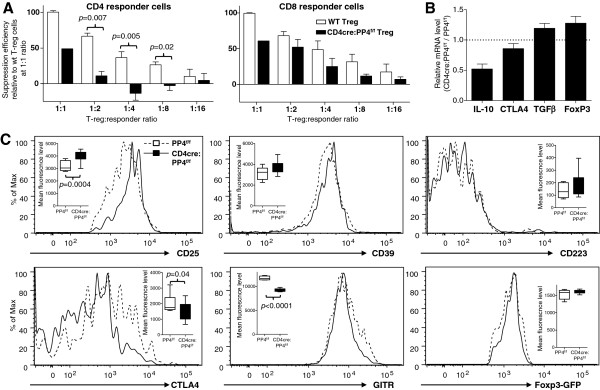
**PP4 deficiency impairs Treg suppression activity. A**, CD4^+^Foxp3-GFP^+^ Treg cells (CD45.1^-^CD90.1^-^) were purified by sorting and co-cultured at titrating ratios with fixed numbers of CFSE-labeled WT responder CD4 and CD8 T cells (CD45.1^-^CD90.1^+^) and irradiated WT APC (CD45.1^+^CD90.1^-^). Division index of CD90.1^+^ CD4 and CD8 responder T cells was calculated from their CFSE patterns on d 3. Treg-mediated suppression was calculated by the division index differences between Treg-added samples and responder-only control. Relative suppression efficiencies were plotted after normalized to that of WT Treg cells at 1:1 ratio (*E* = 3, *n* = 4-5 group except for CD4cre:PP4^f/f^ Treg at 1:1 ratio, for which *n* = 1 due to extremely low number of Foxp3-GFP^+^ cells in the CD4cre:PP4^f/f^ mice). **B**, LN CD4^+^Foxp3-GFP^+^ Treg cells were sorted by flow cytometry. cDNA was synthesized using RNA from these cells and used for qPCR analyses. Relative mRNA level normalized to β actin results are shown (*n* = 2). **C**, LN CD4^+^Foxp3-GFP^+^ Treg cells were analyzed for various Treg markers and Foxp3-GFP expression. Representative plots on gated CD4^+^Foxp3-GFP^+^ cells are shown. Statistical analyses of the mean fluorescence levels are also shown (inserts; *n* = 8 ~ 10). See Additional file 1: Figure S1 for flow cytometry gating strategies.

To examine whether PP4 deficiency also altered the ability of Treg cells to suppress T cell proliferation, WT- or CD4cre:PP4^f/f^-Foxp3-GFP^+^ cells were sorted and co-cultured with activated WT responder T cells. Analyses of the responder cell proliferation showed that CD4cre:PP4^f/f^ Treg cells were significantly less effective in suppressing the proliferation of CD4 responders (Figure 2A, left panel; *p* = 0.007-0.02); the suppression of CD8 responders by CD4cre:PP4^f/f^ Treg cells was also less effective, as indicated by the reduced suppression efficiency at all Treg : responder ratios (Figure 2A, right panel; *p* > 0.05). Since the suppressor functions of Treg cells are thought to be mediated by cytokines such as TGFβ and IL-10, as well as by surface receptors such as CD25, CTLA4 and GITR [9], we thus performed quantitative PCR (qPCR) to examine the expression of these genes. The results showed that PP4-deficient Treg cells exhibited significantly reduced transcription of *il10* and slightly less transcription of *ctla4* (Figure 2B); the mRNA levels of *tgfb1* and *foxp3* were not clearly altered (Figure 2B). Meanwhile, flow cytometry analyses showed that PP4-deficient Treg cells expressed slightly higher level of CD25 (*p* = 0.0004) but normal levels of CD39, CD223, and Foxp3 (Figure 2C); more importantly, the levels of CTLA4 (*p* = 0.04) and GITR (*p* < 0.0001) were both significantly reduced in the CD4cre:PP4^f/f^ Treg cells (Figure 2C). The reduced suppressor function of PP4-deficient Treg cells may thus be attributed to the decreased IL-10, CTLA4, and GITR expressions.

**Figure 3:**
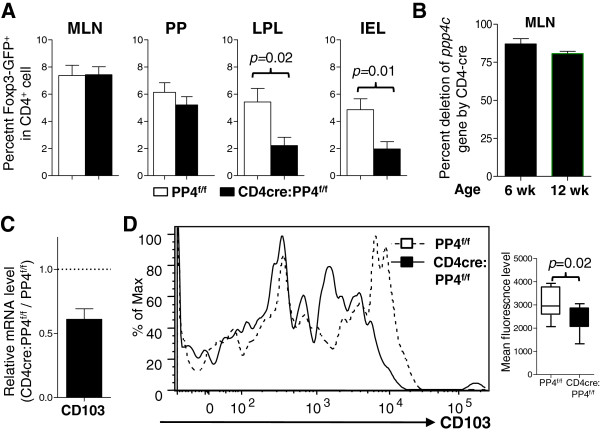
**Defective gut homeostasis of Treg cells in the CD4cre:PP4**^**f/f **^**mice. A**, MLN, Peyer’s patch (PP), LPL, and IEL cells were isolated and analyzed for the percentages of Foxp3-GFP^+^ cells in gated CD4 T cell populations (*n* = 6 ~ 16). **B**, MLN CD4^+^Foxp3-GFP^+^ Treg cells from MLN were MACS-purified from 6 or 12 wk old mice and analyzed for *ppp4c* deletion efficiency as in Figure 1G (*n* = 3). **C**, RNA from sorted LN CD4^+^Foxp3-GFP^+^ cells were analyzed as in Figure 2B. Relative CD103 mRNA levels normalized to β actin results are shown (*n* = 2). **D**, LN CD4^+^Foxp3-GFP^+^ Treg cells were analyzed as in Figure 2C. A representative CD103 plot in gated CD4^+^Foxp3-GFP^+^ population is shown (left panel). Statistical analyses of the mean fluorescence levels are also shown (right panel; *n* = 8 ~ 10). See Additional file 1: Figure S1 for all flow cytometry gating strategies.

In addition to the thymus and spleen, we also found reduced numbers of Foxp3-GFP^+^ Treg cells in the gut of CD4cre:PP4^f/f^ mice: while the percentages of Foxp3-GFP^+^ Treg cells were relatively unchanged in the mesenteric lymph nodes (MLN) and Peyer’s patches, the numbers of Foxp3-GFP^+^ Treg cells in the lamina propria lymphocyte (LPL) (*p* = 0.02) and intra-epithelial lymphocyte (IEL) (*p* = 0.01) subsets were both significantly reduced in the CD4cre:PP4^f/f^ mice (Figure 3A). qPCR of sorted CD4cre:PP4^f/f^ MLN Foxp3-GFP^+^ Treg cells again confirmed the efficient deletion of the *ppp4c* gene in these cells (Figure 3B). That the numbers of PP4-deficient Treg cells were reduced in the LPL and IEL but not in the MLN implicate a potential homing defect of PP4-deficient Treg cells. Indeed, when circulating PP4-deficient Foxp3-GFP^+^ Treg cells in the peripheral LN were analyzed, we found significant reductions in both the transcription (Figure 3C) and surface expression (Figure 3D) of CD103 (α_E_ integrin), which was reported to be important for gut-homing of lymphocytes [23] and Treg-mediated suppression of experimental colitis [24]. Combined with the reduced Treg cell numbers and ineffective suppressor function, our results suggest that PP4 plays a non-redundant role in the differentiation, function, and gut homeostasis of Treg cells.

### Spontaneous rectal prolapse and colitis develop in the CD4cre:PP4^f/f^ mice

**Figure 4:**
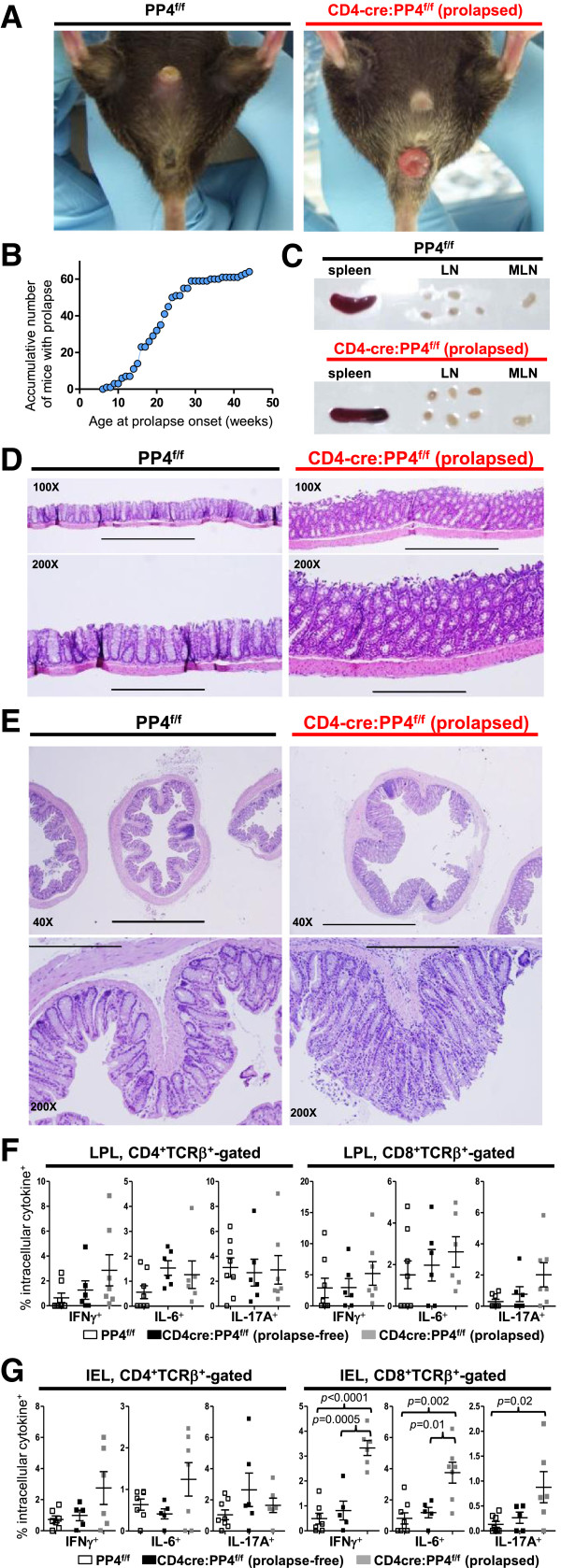
**CD4cre:PP4**^**f/f **^**mice develop spontaneous rectal prolapse and colitis with symptoms resembling Crohn’s disease. A**, Visualization of rectal prolapse in the CD4cre:PP4^f/f^ mice. Results from controls PP4f/f mice are also shown. **B**, Age of prolapse onset in the CD4cre:PP4^f/f^ mice (*n* = 64). **C**, Mild splenomegaly and LN adenopathy in prolapsed CD4cre:PP4^f/f^ mice. Results from controls PP4^f/f^ mice are also shown. **D-E**, Haematoxylin and eosin staining of formaldehyde-fixed longitudinal **(D)** or transverse **(E)** section of normal PP4^f/f^ (left column) or prolapsed CD4cre:PP4^f/f^ colons (right column) are shown. Bars indicate 1000 μm (40X), 400 μm (100X) and 200 μm (200X) respectively. **F-G**, LPL **(F)** and IEL **(G)** cells were isolated and analyzed by flow cytometry for intracellular cytokines. Statistical analysis results on gated CD4^+^ (left panels) or CD8^+^ (right panels) cells are shown (*n* = 5-7). See Additional file 1: Figure S1 for flow cytometry gating strategies.

When maintaining the CD4cre:PP4^f/f^ mouse colony, we found that, by the age of 15 wk old, ~60% of the CD4cre:PP4^f/f^ mice developed spontaneous rectal prolapse (Figure 4A-B) that was rarely (<0.5%) observed in the PP4^f/f^ and Lckcre:PP4^f/f^ mice housed in the same room. The rectal prolapse was accompanied by mild splenomegaly and lymphoadenopathy (Figure 4C) that were occasionally observed in prolapse-free CD4cre:PP4^f/f^ mice. Histological examination of the colons from prolapsed CD4cre:PP4^f/f^ mice showed typical signs of colitis, such as submucosa thickening, epithelial hyperplasia, loss of goblet cells, and mononuclear cell infiltration (Figure 4D-E). To examine if the inflammation also encompassed the small intestine, we isolated IEL and LPL from the small intestines of PP4^f/f^ littermates, prolapse-free CD4cre:PP4^f/f^, and prolapsed CD4cre:PP4^f/f^ mice, and stained them intracellularly for pro-inflammatory cytokines such as IFNγ, IL-6, and IL-17A. No significant difference was found between prolapse-free CD4cre:PP4^f/f^ mice and PP4^f/f^ littermates (Figure 4F-G). In contrast, prolapsed CD4cre:PP4^f/f^ mice exhibited elevated percentages of IFNγ^+^, IL-6^+^ and IL-17A^+^ T cells, particularly in the CD8^+^ IEL compartment (*p* < 0.001 ~ 0.02, Figure 4G), implicating the presence of mucosal inflammation in the small intestine. The gut inflammation in prolapsed CD4cre:PP4^f/f^ mice thus bears a resemblance to human Crohn’s disease by the transmural pathology and the presence of inflammatory T cells in the upper gut [25].

### The spontaneous prolapse is not preceded by accumulation of pro-inflammatory T cells in the gut

**Figure 5:**
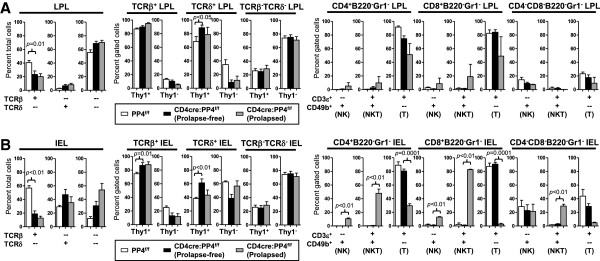
**PP4 deficiency reduces LPL and IEL αβ T cell numbers without significantly impacting their subset distributions. A-B**, LPL **(A)** and IEL **(B)** were isolated and analyzed for the expression of various markers by flow cytometry. Results on total (left panels), TCRβ/TCRδ-gated (middle three panels), or B220^-^Gr1^-^-gated (right three panels) cells are shown (*n* = 2-8). See Additional file 1: Figure S1 for flow cytometry gating strategies.

While the reduced numbers of gut Treg cells (Figure 3A) correlates with the onset of colitis and rectal prolapse, potential alterations in the composition or function LPL an IEL may also contribute. In this regard, we analyzed LPL and IEL cells for the expression of various lymphocyte markers. The results showed that, similar to the spleen and LN, LPL and IEL of prolapse-free CD4cre:PP4^f/f^ mice showed reduction in αβ T cells (Figure 5A-B, left panels); the onset of prolapse had no effect on this reduction (Figure 5A-B, left panels). When further subsetted based on the expression of Thy1 (CD90), we observed a slight increase in the percentages of Thy1^+^TCRδ^+^ LPL and Thy1^+^TCRβ^+^ or Thy1^+^TCRδ^+^ IEL (Figure 5A-B, middle panels). However, the most significant alterations were the accumulation of CD49b^+^ NK/NKT cells and the accompanying reduction of CD3ϵ^+^ T cells in the IEL compartment of prolapsed CD4cre:PP4^f/f^ mice (Figure 5B, right panels). In parallel, we isolated IEL T cells from prolapse-free WT or CD4cre:PP4^f/f^ mice and measured their cytokine secretion to test whether the residual PP4-deficient IEL T cells preferentially secreted inflammatory cytokines. The analyses revealed similar productions of IL-1α, IL-2, IL-6, IL-17A, IFNγ and TNFα between WT and PP4-deficient IEL T cells (Additional file 1: Figure S2B). The reduced number of IEL CD3ϵ^+^ T cells in prolapse-free CD4cre:PP4^f/f^ mice and their normal cytokine productions thus suggest that the ablation of PP4 does not induce the accumulation of pro-inflammatory T cells in the gut to cause colitis onset. Instead, the accumulation of IFNγ^+^, IL-6^+^ and IL-17A^+^ IEL cells in prolapsed CD4cre:PP4^f/f^ mice (Figure 4G) may only occur during the latter phase of colitis pathogenesis.

### PP4-deficient T cells are hypo-responsive to antigen stimulation, ineffective for Th17 polarization, and incapable of inducing experimental colitis

**Figure 6:**
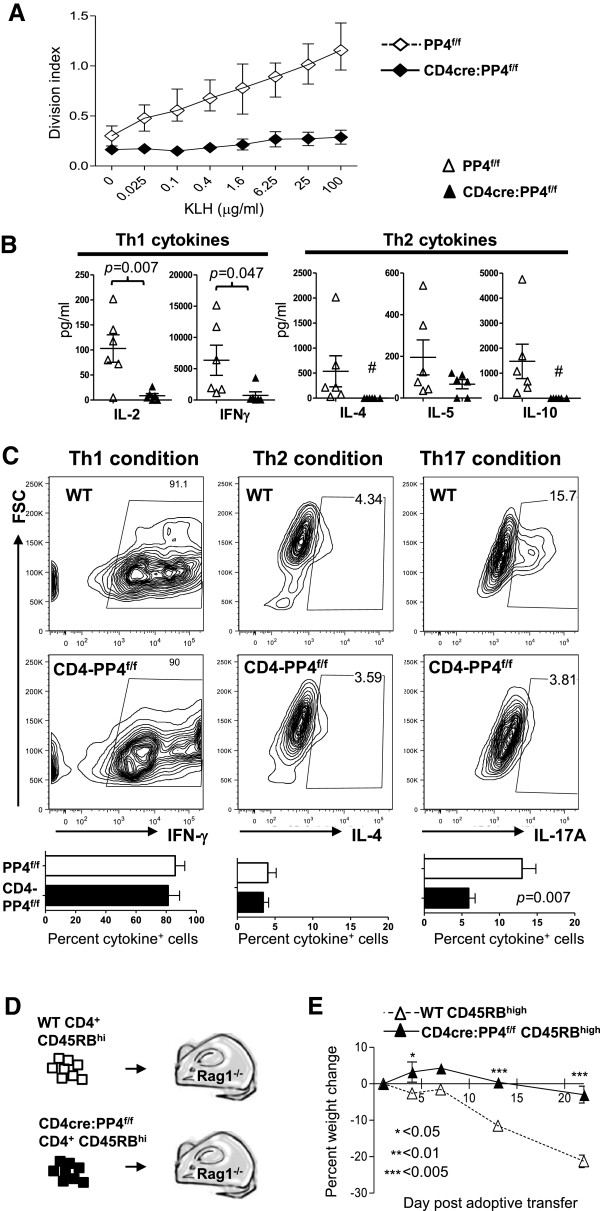
**PP4 deficient T cells are hypo-responsive to antigen stimulation, differentiate into Th17 cells ineffectively, and fail to induce experimental colitis. A-B**, Mice were immunized with KLH/CFA as described in the Materials and Methods. One wk later draining popliteal LN cells were restimulated *in vitro* with KLH; the proliferation of responding T cells **(A)** and their cytokine secretion in culture supernatants **(B)** were assessed on d 3 (*n* = 6). **C**, Naïve T cells were cultured in Th1-, Th2- or Th17-polarizaing conditions for 3 d, followed by intracellular cytokine staining for measuring their polarization efficiency. Representative flow cytometry plots are shown. Statistical analyses results are shown (*n* = 4-7). **D-E**, CD4^+^CD45RB^high^ cells were sorted and transferred (4x10^5^/mouse) into RAG1^-/-^ mice **(D)**. The recipients’ weight changes were monitored for 21 d (*E* = 3, *n* = 5 ~ 6) **(E)**. See Additional file 1: Figure S1 for flow cytometry gating strategies.

We next immunized the CD4cre:PP4^f/f^ mice with keyhole limpet hemocyanin (KLH) to assess whether the deletion of PP4 induced novel hyper-reactive T cells. The results showed that, contrary to the prediction based on the inflammatory nature of colitis, CD4cre:PP4^f/f^ T cells were actually hypo-responsive to restimulation by KLH (Figure 6A). In addition, the secretion of IL-2 and IFNγ was also reduced, while the production of IL-4 and IL-10 were undetectable, in PP4-deficient T cells (Figure 6B). Hinging on these altered cytokine productions, Th1, Th2 and Th17 polarizations were compared between control and CD4cre:PP4^f/f^ T cells. Interestingly, the efficiency of Th1 and Th2 polarization was similar between the two populations; however, PP4-deficient T cells differentiated into IL-17A-secreting Th17 cells with much reduced efficacy (*p* = 0.007, Figure 6C). Finally, to test if the ablation of PP4 enhanced the overall colitogenic ability of peripheral T cells, we adoptively transferred WT or PP4-deficient CD4^+^CD45RB^hi^ cells into RAG1^-/-^ recipients to induce experimental colitis (Figure 6D) [26]. While WT cells successfully induced wasting syndromes in the recipients, PP4-deficient CD4^+^CD45RB^high^ cells failed to do so (Figure 6E); these results could potentially be linked with the hypo-proliferation (Figure 6A) or reduced Th17 polarization (Figure 6C) of PP4-deficient T cells. Regardless, these data argue against the induction of novel colitogenic T cells by PP4 deficiency, and suggest that other factors are responsible for inducing the onset of the spontaneous colitis in these mice.

### The spontaneous colitis in the CD4cre:PP4^f/f^ mice requires commensal bacteria and is heralded by systematic granulocyte infiltration

**Figure 7:**
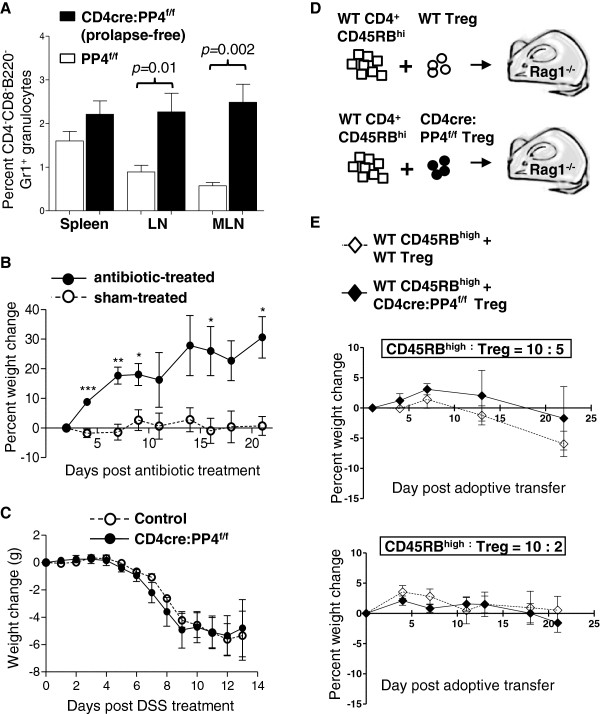
**Analyses of factors that may contribute to the onset of colitis in the CD4cre:PP4**^**f/f **^**mice. A**, Cells from various tissues were stained and analyzed for lymphocyte marker expression. Percentages of CD4^-^CD8^-^B220^-^Gr1^+^ granulocytes in total cells are shown (*n* = 9-12). **B**, Prolapsed CD4cre:PP4^f/f^ mice were treated with antibiotic in drinking water (antibiotic-treated) or left untreated (sham-treated). Percent weight changes of these mice up to 21 d are shown (*E* = 2, *n* = 3). **C**, Control or prolapse-free CD4cre:PP4^f/f^ littermates were treated with 2% DSS in drinking water and monitored daily for weight changes for 13 d. **D-E**, WT CD4^+^CD45RB^high^ cells (4x10^5^/mouse) were purified and co-transferred with sorted WT- or CD4cre:PP4^f/f^-Foxp3-GFP^+^ Treg cells (2x10^5^/mouse, top panel, *n* = 4; 8x10^4^/mouse, bottom panel, *n* = 10) into RAG1^-/-^ mice **(D)**. Recipients’ weight changes were monitored for 21 d **(E)**. See Additional file 1: Figure S1 for all flow cytometry gating strategies.

To gain more insight into the pathophysiological mechanisms of the spontaneous colitis in the CD4cre:PP4^f/f^ mice, we conducted a series of experiments to investigate the associated factors. First, we found that the inflammation in the gut is preceded by the accumulation of CD4^-^CD8^-^B220^-^Gr1^+^ granulocytes in the spleen, LN, and MLN in prolapse-free CD4cre:PP4^f/f^ mice (Figure 7A). When colitic CD4cre:PP4^f/f^ mice were treated with broad-spectrum antibiotics, the colitis pathology and weight loss were effectively reversed (Figure 7B). These data suggest that the spontaneous colitis in the CD4cre:PP4^f/f^ mice may be caused by the failure to contain commensal bacteria in the gut. Treatment of WT and CD4cre:PP4^f/f^ mice with dextran sodium sulfate (DSS), however, caused similar acute colitis symptoms in both mice (Figure 7C), and suggested that PP4 deficiency likely did not directly disrupt the gut barrier to predispose the CD4cre:PP4^f/f^ mice to mucosal inflammation. Lastly, to test whether the functional defect in CD4cre:PP4^f/f^ Treg cells was the predominant cause for the spontaneous colitis in the CD4cre:PP4^f/f^ mice, we co-transferred WT or CD4cre:PP4^f/f^ Treg cells with WT CD4^+^CD45RB^high^ T cells into RAG1^-/-^ recipients (Figure 7D). Surprisingly, the results showed that CD4cre:PP4^f/f^ Treg cells were still capable of suppressing the induction of experimental colitis (Figure 7E, top panel) despite their reduced *in vitro* suppressor activity (Figure 3A). Reducing the number of co-transferred Treg cells yielded similar outcomes (Figure 7E, bottom panel). Together, these data suggest that the onset of spontaneous colitis in the CD4cre:PP4^f/f^ mice cannot be attributed solely to the functional defects of Treg cells, but is likely orchestrated by additional factors, such as the gut homeostasis of Treg cells, the infiltration of commensal bacteria, and the activation of innate immunity.

## Discussion

In this report, we have described the spontaneous prolapse and colitis in mice with T cell-specific ablation of PP4, the CD4cre:PP4^f/f^ mice. What are the factors that might have contributed to the onset of colitis in these mice? Altered Treg cell functions (Figure 2) are likely a significant factor due to their immune-regulatory roles. Commensal bacteria are clearly a prerequisite, as indicated by the amelioration of colitis following antibiotic treatment (Figure 7B). The accumulation of granulocytes in prolapse-free CD4cre:PP4^f/f^ mice (Figure 7A) and the increased number of pro-inflammatory IEL T cells in prolapsed CD4cre:PP4^f/f^ mice (Figure 4G) further implicate the involvement of innate and adaptive, respectively, anti-commensal immune responses in the gut. Finally, the seemingly contradictory hypo-responsiveness of PP4-deficient T cell to antigen stimulation (Figure 6A-B) may actually contribute to the onset of colitis by preventing the clearance of infiltrating commensal bacteria. While it is difficult to quantify the relative contributions of the individual factors, these observations are consistent with the core scenario that uncontrolled activation of mucosal innate and adaptive immune cells, caused by defective Treg suppression, results in persistent, commensal-dependent gut inflammation that eventually breaches the mucosal barrier for the onset of colitis in the CD4cre:PP4^f/f^ mice.

Although our data fit nicely into this working model, the clear disparity in the colitis incidence rates of Lckcre:PP4^f/f^ (<0.5%) and CD4cre:PP4^f/f^ (>60%) mice needs to be addressed. In this regard, a major difference between these mice is that the Lckcre:PP4^f/f^ mice exhibit severe lymphopenia [2], while the CD4cre:PP4^f/f^ mice contain reduced but substantial number of peripheral T cells (Figure 1B-C). The residual PP4-deficient T cells in the CD4cre:PP4^f/f^ mice may thus be essential for the T cell component of the inflammatory response and tissue damage during the late phase of colitis pathogenesis. Without the induction of aggravated T cell inflammatory responses, innate immunity may be able to keep the commensal bacteria in check to prevent excessive tissue damage. Such a scenario has been observed when comparing the colitis incidence between TCRα^-/-^, TCRβ^-/-^ and RAG1^-/-^ mice housed in specific pathogen-free facility [27]. Alternatively, it is possible that the difference in the timing of the deletion of the *ppp4c* gene may allow the generation of colitogenic cells in the CD4cre:PP4^f/f^ thymus but not in the Lckcre:PP4^f/f^ thymus. One such candidate is NKT cells, whose maturation begins during the DN-DP transition and is completed at the DP stage [28] However, we did not find any significant alteration in the percentages of CD3ϵ^+^CD49b^+^ NKT cells in gated CD4^+^ and CD8^+^ populations in the CD4cre:PP4^f/f^ mice (Additional file 1: Figure S2C), although they did accumulate in the gut of prolapsed animal (Figure 5B). Results from IEL T cell analyses in prolapse-free CD4cre:PP4^f/f^ mice (Figure 5 and S2B), from helper T cell polarization (Figure 6C), and from the induction of experiment colitis (Figure 6E) also helps rule out the possibility that PP4 deficiency induces novel pro-inflammatory, colitogenic effector CD4 T cells. Nevertheless, such a possibility remains viable for other T cell subsets.

Treg deficiency caused by the ablation of PP4 is fairly broad, encompassing defects in nTreg/iTreg differentiation, suppressor functions and gut homeostasis. In this context, PP4 may mediate these diverse effects either by functioning through a single master factor that regulates a complex network of downstream genes, or by acting individually on multiple target proteins to impact various signaling pathways, or both. For the former, monomeric regulation, the primary candidate that may be regulated by PP4 is Foxp3. Evidence supporting a potential role of PP4 comes indirectly from recent reports showing that the stability of Foxp3 is reduced when its serine 19 residue is phosphorylated by CDK2 [29], and that the activity of Foxp3 is down-regulated when its serine 418 residue is dephosphorylated by protein phosphatase 1 [30]. However, in either case the constitutive phosphorylation of these residues should enhance Foxp3 activity, yet our data indicate that the loss of PP4 is manifested in the form of defective Treg functions. In this regard, Foxp3 contains several other potential serine/threonine phosphorylation sites on residues 13, 25, 114, 137 and 141 [31] that may serve as the target of PP4-mediated dephosphorylation for the regulation of Foxp3 activity. Alternatively, the Ikaros family transcription factors, Eos and Helios, have been shown to regulate the transcription [32] or activity of Foxp3 [33], respectively. Our observation that the transcription and expression of Foxp3 are not significantly altered in PP4-deficient Treg cells (Figure 3B-C) argues against a dominant role for Helios, but potential PP4-mediated regulation on Eos remains a possibility.

Other than Foxp3, the reduced Treg cell numbers may also be caused by defective Treg cell survival or expansion in the absence of PP4. This possibility is supported by our previous report showing that the deletion of PP4 induces apoptosis in developing thymocytes [2] and by the recent findings that the inhibition of PP4 blocks cell cycle progression [34]. Alternatively, recent reports suggest that Treg cells require proper TCR activation to achieve optimal differentiation and homeostasis [35,36]. Since PP4 is shown to be involved in TCR signaling [2] and NFκB activation [37], altered TCR activation may also contribute to the Treg defects in the CD4cre:PP4^f/f^ mice. We are currently investigating these possibilities.

## Conclusions

In this report, we have described the defects in Treg differentiation, function and homeostasis caused by PP4 deficiency. These defects are associated with altered IL-10, CTLA4, GITR and CD103 expression in PP4-deficient Treg cells, and are accompanied by gut inflammation and spontaneous colitis in the CD4cre:PP4^f/f^ mice. While the molecular mechanisms of PP4-mediated regulations on Treg cells remain to be elucidated, we believe that our characterizations of the CD4cre:PP4^f/f^ mice provide important frameworks for future studies on how PP4, and potentially other phosphatases, may regulate Treg functions and gut immunity.

## Methods

### Mice

PP4^f/f^[2], CD4cre [19], Foxp3-GFP [20] and RAG1^-/-^[38] mice have been described. CD90.1 and CD45.1 C57/Bl6 congenic mice were obtained (Jackson laboratory). PP4^f/f^ mice were crossed with CD4cre mice to generate the CD4cre:PP4^f/f^ mice with T cell-specific deletion of the *ppp4c* gene. CD4cre:PP4^f/f^ mice were further crossed with Foxp3-GFP mice to generate the CD4cre:PP4^f/f^:Foxp3-GFP mice. All mice were housed under specific pathogen-free condition at the Laboratory Animal Center of the National Health Research Institutes (NHRI). Mice with a loss of >20% body weight were removed by euthanasia. All animal experimental procedures followed the guidelines approved by the NHRI Institutional Animal Care and Use Committee.

### Antibodies and flow cytometric analysis

Antibodies against mouse epitopes of B220, CD3ϵ, CD4, CD8, CD11b, CD25, CD39, CD45RB, CD49b, CD62-L, CD90, CD223, CTLA4, CXCR5, GITR, Gr1, TCRβ, TCRδ, TER119, IL-4, IL-6, IL-17A and IFNγ conjugated with various fluorescent dyes or biotin, 7AAD and AnnexinV-APC (all purchased from BioLegend or BD Biosciences) were used for surface and intracellular staining following standard protocols. CFSE (Invitrogen) was loaded into targets cells following the manufacturer’s suggestions. Flow cytometry results were obtained on 8-color FACSCanto II with FACSDiva software (BD Biosciences), were and analyzed by FlowJo software (Tree Star).

### PCR and qPCR

For estimating the efficiency of *ppp4c* gene deletion, genomic DNA was extracted from sorted primary cells with standard protocols. Oligonucleotides for qPCR of exon 2 were 5′-GGGCGGTCCCAGAATCGAGT-3′ (primer a) and 5′-ATCAGCTCGCAGCGCCGTAG-3′ (primer b). For exon3, the oligonucleotides used were 5′-CCAGTTGGCAACAAGGAGCCAT-3′ (primer c) and 5′-CCAGCCCAATTCCTGACCTT-3′ (primer d) (see Figure 1G for primer locations). For gene transcription, total RNA was extracted from sorted LN CD4^+^Foxp3-GFP^+^ cells and converted into cDNA with standard protocol. The primers used are: Actin-1: 5′ AAGTGTGACGTTGACATCCGTAA-3′; Actin-2: 5′- TGCCTGGGTACATGGTGGTA-3′. CD103-1: 5′-CGTGGAGAAGAAGGCAGAGT-3′; CD103-2: 5′-TCGGGGGTAAAGGTCATAGAT-3′; CTLA4-1: 5′-CTCAACTGCAGCTGCCTTCTAGGA-3′; CTLA4-2: 5′-AAGCTGGCGACACCATGGCT-3′; Foxp3-1: 5′-GGCCCTTCTCCAGGACAGA-3′; Foxp3-2: 5′-GCTGATCATGGCTGGGTTGT-3′; IL-10-1: 5′-TGCAGGACTTTAAGGGTTACTTGGG-3′; IL-10-2: 5′-CCTTGCTCTTATTTTCACAGGGGAG-3′; TGFβ-1: 5′-GCTCGCTTTGTACAACAGCACCC-3′; TGFβ-2: 5′-GCTTCCCGAATGTCTGACGTATTG-3′; qPCR was performed using FastStart Universal Probe Master Rox (Roche Applied Science) on Realplex4 with Mastercycler ep realplex software (Eppendorf). Genotyping PCR for the CD4cre transgene was performed with oligonucleotides 5′-TCTCTGTGGCTGGCAGTTTCTCCA-3′ and 5′-TCAAGGCCAGACTAGGCTGCCTAT-3′. Genotyping of the PP4^f^ allele was performed with oligonucleotides 5′-TGCTCTGGTGCAGGAGATGTGTG-3′, 5′-ACGTGATTTGCGAAAGCCTCTCA-3′, and 5′-CTTGGTAGAAGAGAGCAACGTGCAG-3′ in a three-primer reaction. PCR conditions are available upon request.

### Cell sorting and culture

For qPCR, Treg suppression assays and adoptive transfer, cells were stained for surface markers and sorted on FACSAria (BD Biosciences) or enriched by magnetic-assisted cell sorting (MACS). All primary cells were cultured in DMEM supplemented with 1 x non-essential amino acid, 2 mM L-glutamine, 2 mM Glutamax, 1 mM sodium pyruvate, 10 mM HEPES (all from Invitrogen), 10% FBS, 100 U/ml penicillin, 100 mg/ml streptomycin (all from Biological Industries) and 125 μM 2-mercaptoethanol (Sigma-Aldrich).

### Histological analyses

Colons were excised, flushed with PBS, and fixed in 10% formaldehyde for 1 hr before embedded in paraffin. Longitudinal or transverse sections were cut and stained with haematoxylin and eosin with standard protocols. Histological images were obtained on Olympus IX71 microscope with Olympus DP70 camera using Olympus DP controller software (Olympus).

### Isolation of IEL and LPL cells

Small intestines were harvested and flushed with CMF solution (containing 2% FBS, 10 mM HEPES, Ca^2+^/Mg^2+^-free HBSS) before removing the Peyer’s patches. Residual small intestine was cut into 0.5 cm pieces and washed six more times with CMF solution, incubated in 10% FBS/0.1 mM EDTA/CMF at 37°C for 15 min with rotary shaking (220 rpm), transferred to a fresh tube, and vortexed for 15 sec at maximum setting. After the tissues settled, supernatant was saved in a fresh tube. The precipitated tissues were re-applied in the above incubation/transfer procedure for four more times. All supernatants were pooled for IEL and epithelial cells isolation via Percoll gradient separation. The remaining intestine pieces were washed four times with 10% FBS/5 mM EDTA/CMF solution at 37°C for 15 min with rotary shaking (220 rpm). After the last wash, the intestine pieces were incubated in 10% FBS/ RPMI containing 100 U/ml type VIII collagenase (Sigma-Aldrich) for 2 hr at 37°C with rotary shaking (220 rpm) and media change at 1 hr. The debris was allowed to settle, and the resulted supernatant was subjected to Percoll gradient separation for the isolation of LPL cells. Percoll (Sigma-Aldrich) gradient separation (for IEL: 44%/67%; for LPL: 40%/100%) was performed by loading the supernatant atop of appropriate Percoll gradients, followed by centrifugation at 400g for 20 min and collection of IEL or LPL cells at the interface.

### Experimental colitis induction and antibiotics treatment

For adoptive transfer-induced experimental colitis, CD4 T cells were enriched from total splenocytes by MACS negative selection for B220, CD11b, CD49b, CD8, and Ter119. CD4^+^CD45RB^high^ (upper 40% of CD45RB^+^ cells) or CD4^+^CD25^+^Foxp3-GFP^+^ cells were purified from these cells by sorting. Sorted cells were then transferred via tail vein into RAG1^-/-^ recipients as indicated in the figure legend. For DSS-induced colitis, mice were administered 2% DSS dissolved in sterilized drinking *ad libitum* for 14 d. Animals were weighed daily and monitored for rectal bleeding, diarrhea, and general signs of morbidity. For antibiotics treatment, mice received drinking water containing 0.66 mg/ml ciprofloxacin, 2.5 mg/ml metronidazole (Sigma-Aldrich) and 1.5% fructose (to encourage consumption) for 3 weeks. Control animals were given drinking water containing 1.5% fructose only. Both DSS water and antibiotic solution were replaced 2-3 times weekly.

### KLH immunization, T cell response and cytokine measurement

Mice at 6-8 wk age were immunized in the hind footpad with 0.1 ml of 1:1 emulsion of CFA (Difco) and 1 mg/ml KLH (Sigma-Aldrich). One wk later draining popliteal LN cells were harvested, labeled with CFSE, and restimulated with titrating doses of KLH *in vitro* for 3 d. The proliferation of responding cells was then measured by CFSE dye-dilution, while the cytokine production was assessed with FlowCytomix Mouse Th1/Th2 10plex kit (eBioscience) following the manufacturer’s procedure. Cytokine production from isolated IEL cells was assessed similarly.

### Treg/Th1/Th2/Th17 polarization and suppression assays

For *in vitro* polarization of Treg cells, naïve CD4^+^CD62-L^+^ cells were purified by MACS from splenocytes and stimulated with 1.6 μg/ml soluble anti-CD28 and plate-bound anti-CD3ϵ in the presence of 5 ng/ml TGFβ, 10 μg/ml anti-IL-4 and 10 μg/ml anti-IFNγ for 3 d. Cells were fixed in 4% paraformaldehyde/PBS prior to surface staining and flow cytometry analyses with standard protocols. Th1 (5 ng/ml IL-2, 10 ng/ml IL-12 and 10 μg/ml anti-IL-4), Th2 (10 ng/ml IL-2, 4 ng/ml IL-4, 10 μg/ml anti-IFNγ and 10 μg/ml anti-IL-12) and Th17 (30 ng/ml IL-6, 1 ng/ml TGFβ, 10 μg/ml anti-IFNγ and 10 μg/ml anti-IL-4) cells were polarized and assessed similarly. For Treg suppression assays, CD4 T cells were enriched from pooled spleen and LN cells by MACS. CD4^+^Foxp3-GFP^+^ Treg cells were then purified from these cells by sorting. Irradiated APC were prepared from C57Bl/6 splenocytes following red blood cell lysis and 200 Gray irradiation. WT responder T cells were prepared from pooled spleen and LN cells from CD90.1 congenic mice by MACS, and were loaded with CFSE. Cell culture was set up in 96-well U-bottomed plates with 1 μg/ml soluble-anti-CD3ϵ at a final volume of 200 μl, and contained 5 × 10^4^ WT responder cells, 2 × 10^5^ irradiated APC, and titrating number of Treg cells to obtain 1:1 to 16:1 ratio of responder : Treg cells. The proliferation of WT responder T cells were assessed on d 3 by flow cytometry; division index was calculated using the FlowJo software (see Additional file 1: Figure S2D for more detail).

### Statistical analyses

When applicable, data were plotted as mean ± SEM with the *p*-values calculated using unpaired two-tailed Student’s *t*-test.

### Availability of supporting data

Gating strategies of flow cytometry analyses and additional data are available as online supplemental materials in Additional file 1.

CD4cre, CD4 promoter-driven Cre recombinase transgene CD4SP, CD4 single-positive; DSS, dextran sulfate sodium; *E*, number of independent experiment; IBD, inflammatory bowel disease; IEL, intra-epithelial lymphocyte; KLH, keyhole limpet hemocyanin; Lckcre, Lck proximal promoter-driven Cre recombinase transgene; LPL, lamina propria lymphocyte; LN, lymph node; MACS, magnetic-assisted cell sorting; MLN, mesenteric lymph node; NHRI, National Health Research Institutes; PP4, protein phosphatase 4; qPCR, quantitative PCR; Treg, regulatory T.

## Competing interests

The authors declare no financial or non-financial competing interest.

## Authors’ contributions

CH designed and executed experiments as well as prepared the manuscript. FL designed and executed experiments and analyzed data. JS generated the CD4cre:PP4^f/f^ mice and discovered the prolapse phenotype. EH, WH, YL, and YC executed experiments. TT generated the CD4cre:PP4^f/f^ mice, discovered the prolapse phenotype and designed experiments. All authors read and approve the final manuscript.

## Supplementary Material

Additional file 1: Figure S1 Flow cytometry gating strategies. **A**, Gating strategies for Figure 1A and 1C. **B**, Gating strategies for Figure 1B. **C**, Gating strategies for Figures 1D, 2C, 3A and 3D. **D**, Gating strategies for Figure 1E. **E**, Gating strategies for Figures 1F and 6C. **F**, Gating strategies for Figure 2A and 6A. G, Gating strategies for Figure 5A and 5B. H, Gating strategies for Figure 4F and 4G. **Figure S2.** Additional supporting results. **A**, Peripheral T cells were purified by MACS, followed by western analyses for the expression of PP4. Representative results from two experiments are shown. Actin loading control is also shown. **B**, IEL cells were purified as in the Materials and Methods and activated by 1.6 mg/ml plate-bound anti-CD3e and anti-CD28 for 3 d. Culture supernatants were then analyzed for the secretion of cytokines. **C**, Splenocytes from control or prolapse-free CD4cre:PP4f/f mice were stained the respective markers and analyzed for the percentage of CD4-CD8-B220-Gr1+ granulocytes (n=9-11). D, Calculation of division index for the CFSE dye-dilution assay. In this example, the generation numbers (0-6) are marked by the CFSE peaks. The total number of cell division and the total number of starting cells represented by all the generations are then calculated to obtain their ratio as the division index. See the figure for more details. Click here for file
