# Supplementary material for: Protein phosphatase 4 is an essential positive regulator for Treg development, function, and protective gut immunity
Source: Cell Biosci. 2014 May 7;4:25. doi: 10.1186/2045-3701-4-25 (PMC4045899; doi:10.1186/2045-3701-4-25)
Supplement: Additional file 1: Figure S1 — Flow cytometry gating strategies. A, Gating strategies for Figure 1A and 1C. B, Gating strategies for Figure 1B. C, Gating strategies for Figures 1D, 2C, 3A and 3D. D, Gating strategies for Figure 1E. E, Gating strategies for Figures 1F and 6C. F, Gating strategies for Figure 2A and 6A. G, Gating strategies for Figure 5A and 5B. H, Gating strategies for Figure 4F and 4G. Figure S2. Additional supporting results. A, Peripheral T cells were purified by MACS, followed by western analyses for the expression of PP4. Representative results from two experiments are shown. Actin loading control is also shown. B, IEL cells were purified as in the Materials and Methods and activated by 1.6 mg/ml plate-bound anti-CD3e and anti-CD28 for 3 d. Culture supernatants were then analyzed for the secretion of cytokines. C, Splenocytes from control or prolapse-free CD4cre:PP4f/f mice were stained the respective markers and analyzed for the percentage of CD4-CD8-B220-Gr1+ granulocytes (n=9-11). D, Calculation of division index for the CFSE dye-dilution assay. In this example, the generation numbers (0-6) are marked by the CFSE peaks. The total number of cell division and the total number of starting cells represented by all the generations are then calculated to obtain their ratio as the division index. See the figure for more details. [file 2045-3701-4-25-S1.pdf]

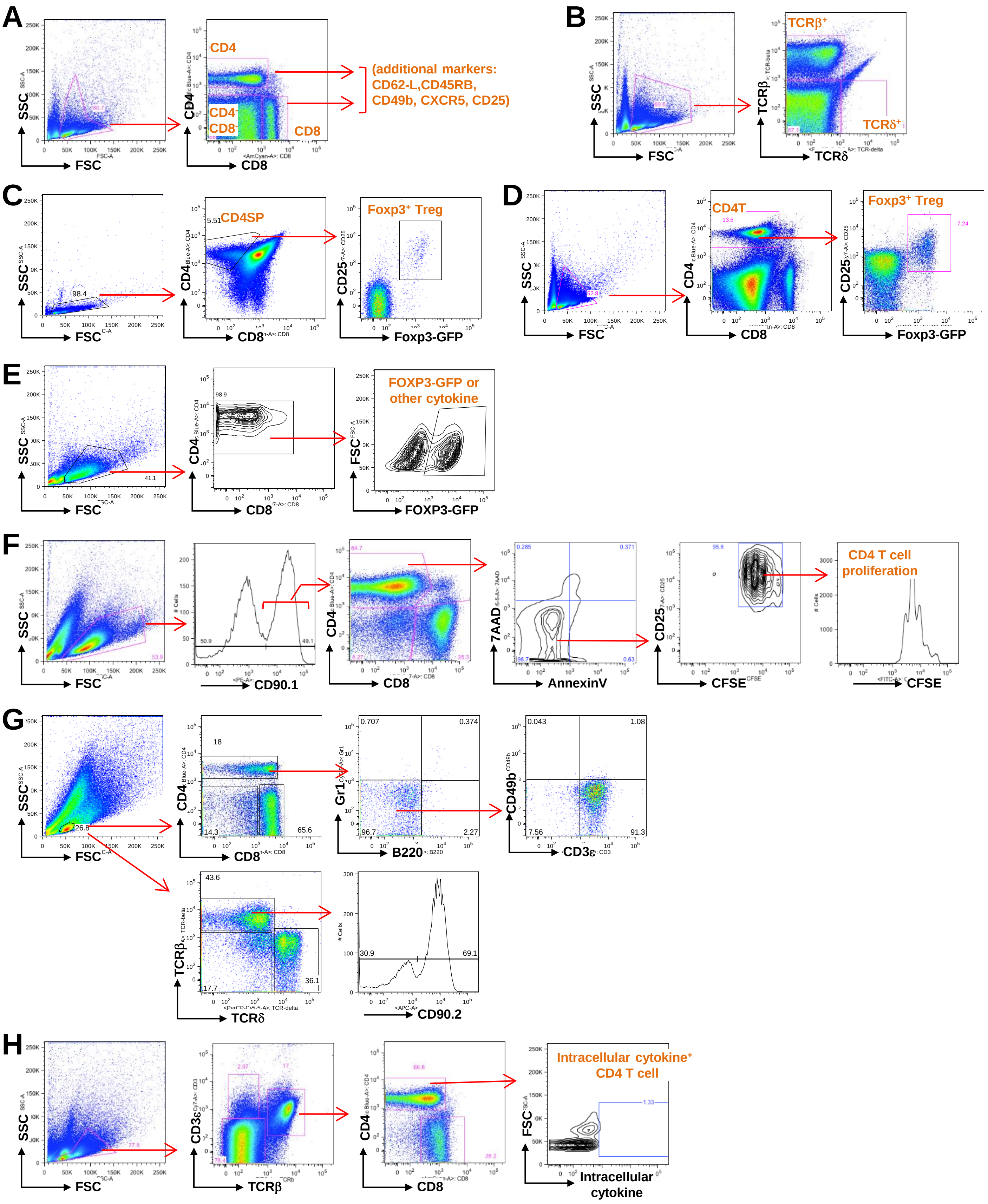

Additional file 1 Figure S1 Flow cytometry gating strategies. **A**, Gating strategies for Figure 1A and 1C. **B**, Gating strategies for Figure 1B. **C**, Gating strategies for Figure 1D, 2C, 3A and 3D. **D**, Gating strategies for Figure 1E. **E**, Gating strategies for Figure 1F and 6C. **F**, Gating strategies for Figure 2A and 6A. **G**, Gating strategies for Figure 5A and 5B. **H**, Gating strategies for Figure 4F and 4G.

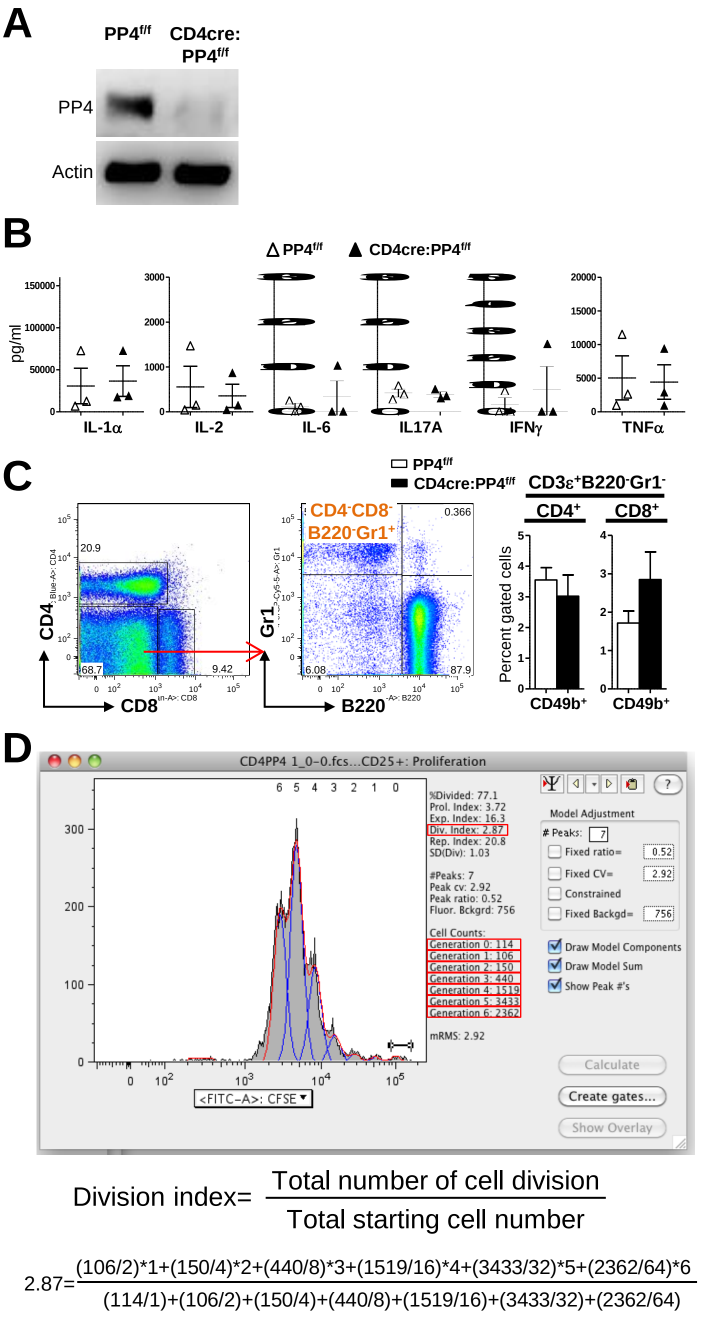

Additional file 1 Figure S2. **A,** Peripheral T cells were purified by MACS, followed by western analyses for the expression of PP4. Representative results from two experiments are shown. Actin loading control is also shown. **B**, IEL cells were purified as in the Materials and Methods and activated by 1.6 μg/ml plate-bound anti-CD3ε and anti-CD28 for 3 d. Culture supernatants were then analyzed for the secretion of cytokines. **C**, Splenocytes from control or prolapse-free CD4cre:PP4$^{f/f}$ mice were stained the respective markers and analyzed for the percentage of CD4^{-}CD8^{-} B220^{-}Gr1^{+} granulocytes (n=9-11). **D**, Calculation of division index for the CFSE dye-dilution assay. In this example, the generation numbers (0-6) are marked by the CFSE peaks. The total number of cell division and the total number of starting cells represented by all the generations are then calculated to obtain their ratio as the division index. See the figure for more details.
